# Live long and prosper: durable benefits of early-life care in banded mongooses

**DOI:** 10.1098/rstb.2018.0114

**Published:** 2019-02-25

**Authors:** Emma I. K. Vitikainen, Faye J. Thompson, Harry H. Marshall, Michael A. Cant

**Affiliations:** 1 Centre for Ecology and Conservation, University of Exeter, Penryn Campus, Penryn, UK; 2 Organismal and Evolutionary Biology Research Programme, Faculty of Biological and Environmental Sciences, University of Helsinki, Helsinki, Finland; 3 Department of Life Sciences, University of Roehampton, London, UK

**Keywords:** early-life effects, cooperative breeding, inclusive fitness, lifetime reproductive success, selective disappearance, social evolution

## Abstract

Kin selection theory defines the conditions for which altruism or ‘helping’ can be favoured by natural selection. Tests of this theory in cooperatively breeding animals have focused on the short-term benefits to the recipients of help, such as improved growth or survival to adulthood. However, research on early-life effects suggests that there may be more durable, lifelong fitness impacts to the recipients of help, which in theory should strengthen selection for helping. Here, we show in cooperatively breeding banded mongooses (*Mungos mungo*) that care received in the first 3 months of life has lifelong fitness benefits for both male and female recipients. In this species, adult helpers called ‘escorts’ form exclusive one-to-one caring relationships with specific pups (not their own offspring), allowing us to isolate the effects of being escorted on later reproduction and survival. Pups that were more closely escorted were heavier at sexual maturity, which was associated with higher lifetime reproductive success for both sexes. Moreover, for female offspring, lifetime reproductive success increased with the level of escorting received *per se*, over and above any effect on body mass. Our results suggest that early-life social care has durable benefits to offspring of both sexes in this species. Given the well-established developmental effects of early-life care in laboratory animals and humans, we suggest that similar effects are likely to be widespread in social animals more generally. We discuss some of the implications of durable fitness benefits for the evolution of intergenerational helping in cooperative animal societies, including humans.

This article is part of the theme issue ‘Developing differences: early-life effects and evolutionary medicine’.

## 1. Introduction

Social evolution theory aims to understand and predict how natural selection acts on heritable social traits, that is, traits that affect the fitness of other members of a population. Hamilton's [1,2] inclusive fitness theory defined the condition (*rb* *>* *c,* known as Hamilton's rule) for which selection can favour the evolution of altruism (i.e. a trait that boosts the lifetime fitness *b* of a recipient, related by coefficient *r*, at a lifetime fitness cost *c* to the actor) directed towards genetic relatives. Subsequent theory has emphasized repeated interactions, intergroup competition and group augmentation as promoters of cooperative behaviour [3–5]. Inclusive fitness theory in particular has provided a very general framework to understand variation in social traits (both behavioural and life-history traits), and to identify ecological and demographic factors that facilitate cooperation and the formation of animal societies [6,7].

Cooperative animal societies, in which ‘helpers’ work to rear offspring that are not their own, are a rich testing ground for these theories because they feature conspicuous examples of altruism or ‘helping’, together with the possibility of measuring the fitness consequences of variation in helping effort and life-history decisions. In addition, research on cooperative vertebrates provides a potentially informative comparator for *Homo sapiens,* one of the few cooperatively breeding primates [8,9]. There is now considerable evidence that major features of human life history (e.g. long period of offspring dependency, short inter-birth interval, early reproductive cessation, prolonged post-reproductive lifespan) have been moulded via kin selection operating in the family groups of our Pleistocene ancestors [10–15].

Although the costs and benefits in Hamilton's rule are in the currency of lifetime direct fitness, tests of kin selection theory and other proposed mechanisms of cooperation (such as reciprocity and coercion [16,17]) rely on measuring reasonable proxies for lifetime fitness impacts. For example, the fitness benefit conferred by helpers might be tested by comparing the number of surviving offspring produced by reproductives with and without the assistance of helpers [11,18,19]. However, the literature on early-life effects and developmental plasticity shows that there may often be delayed impacts of investment that are manifested long after the initial act. In social insects, for example, variation in provisioning in the larval period triggers developmental switches and leads to permanent behavioural and morphological castes [20,21]. In vertebrates, permanent castes are typically lacking, but research on laboratory rodents and humans shows that postnatal care can have lifelong effects on cognitive function, social behaviour and health [22–24]. Thus, the effects of help on a recipient's fitness, particularly when the recipient is an individual offspring, may be manifested long after the helping act itself—even after the helper has died or dispersed.

The potential for early-life investment to ‘programme’ an offspring's subsequent life history could promote or inhibit selection for helping, depending on whether helped offspring are more or less likely to disperse, and more or less likely to produce surviving offspring themselves. These delayed impacts of help represent an ‘internal’ durable benefit conferred by the helper, similar to the ‘external’ durable benefits that can arise through niche construction, for example, the construction of a nest or shelter that benefits future generations. Recent theory suggests that the potential for helping to result in benefits that are manifested in the future (in addition to, or instead of, fitness benefits that are manifested contemporaneously with the helping act) has a strong influence on selection for altruism in structured populations [25]. In these ‘patch-structured’ or ‘group-structured’ models, helping boosts the fecundity (number of offspring) of the local group of kin, but also increases competition among these local kin. The former inclusive fitness benefit of helping is counteracted by the latter inclusive fitness cost resulting from increased competition. The further into the future the benefits of helping are realized, the lower the relatedness of the actor to the individuals in the patch that suffer the costs of competition, and hence the greater the overall strength of selection for helping [25].

The potential durable benefits of helping in cooperative animal societies have been little explored empirically. One exception is Russell *et al*.'s [26] study of meerkats (*Suricata suricatta*), which showed that female offspring that gain most weight during the helping period (and hence are likely to have received more help), and those that are experimentally fed, are more likely to reproduce at some point in their lives and more likely to attain the position of dominant breeder. In other cooperatively breeding vertebrates (including humans), measuring delayed or lifelong impacts of help is challenging because it requires following the recipients of care across their entire lifespan, and recipients often die or disperse before attaining reproductive status.

Here, we investigate the immediate and lifelong consequences for the recipients of helping in a cooperatively breeding mammal, the banded mongoose (*Mungos mungo*), using a 17-year dataset. This species exhibits an unusual form of one-to-one early-life offspring care called ‘escorting’ which provides an opportunity to tease apart genetic, maternal and alloparental effects on development and later life history [27,28]. Multiple females give birth in each breeding attempt, usually on the same day [29], and the communal litter is kept underground for the first month of life. Mothers show no discrimination during suckling, and pups are sometimes observed to move from female to female to suckle [30,31]. From the time that pups emerge from the den until they reach nutritional independence at three months old, pups form exclusive one-to-one caring relationships with adult helpers (their ‘escorts’) who are no more closely related than a random group member [27]. Escorts provision and groom the pups in their care, and carry them away from danger. However, there is great variation among offspring in the amount of escorting received: some pups spend all day every day with their escort, whereas others have to fend for themselves from an early age [27,28].

The escort system allows us to quantify the amount of postnatal help received by individual offspring in each communal litter. By contrast, in most other cooperatively breeding insects, birds and mammals, helper effort is shared across entire litters or broods [32], so it is more difficult to isolate the fitness impacts of the investment by an individual helper on an individual recipient. In addition, our system is unusual because dispersal away from the study site is rare [33,34], and we can follow individuals across their entire lives, from pup to reproducing adult. In this paper, we capitalize on this system to test whether the care received by pups in the first three months of life has lasting effects on their survival and reproduction as adults, long after the period of care has ended.

## 2. Material and methods

### (a) Study species and population

Banded mongooses are small (1.5 kg) cooperatively breeding carnivorous mammals common to sub-Saharan Africa. Since 1995, we have continuously studied a habituated population of wild banded mongooses living on and around the Mweya Peninsula in Queen Elizabeth National Park, western Uganda (0°12′ S, 29°54′ E); for details of the field site and the population, see [35] and references therein. At any one time, the population consists of 8–12 mixed sex groups of 10–30 individuals, plus offspring. On average, four females give birth in each breeding attempt, synchronizing birth to the same day in 64% of breeding attempts [29]. The resulting mixed-parentage litter of pups is guarded at the den during the first month of life by one or more babysitters [35]. After emergence care is provided by escorts up to the age of three months [27,36]. Individuals reach sexual maturity at around 1 year old, and life expectancy at this age is around 3 years (males = 42 months; females = 38 months). There is no reproductive suppression among females in this species: adult females start breeding when they are 1 year old and produce up to four litters per year until they die [33]. Males, by contrast, form an age-based social queue in which the oldest two or three individuals mate-guard and aggressively monopolize access to oestrous females [37,38]. Younger males, though sexually mature, are typically excluded from reproduction until they reach relatively advanced ages (3+ years; [38]).

We collected data from individuals from 12 social groups of on average 22 adult individuals (s.d. 7.3, range 7–37) inhabiting the study area between the years 2000 and 2016. All mongooses in the study population are individually marked using either unique hair-shave patterns or colour-coded collars, and are habituated to close observation from at least 5 m. Additionally, each mongoose is marked with a transponder chip (Wyre Micro Design, UK) or, before the year 2009, with a unique tattoo on the inside of the leg. One or two mongooses in each group are fitted with a radio collar weighing 26–30 g (Sirtrack Ltd, Havelock North, New Zealand) to allow the groups to be located.

### (b) Life-history parameters and genotyping

Over the 17-year study period, each group was visited for at least 20 min every 1–3 days to record the presence and absence of individuals in each group. As banded mongooses almost always disperse in groups, either voluntarily or through a process of violent eviction [39–41], we could distinguish between dispersal and deaths as cause for permanent absence from the group. For the dataset used in the analyses, we included only those individuals whose date of birth and death were both known with at least one week's accuracy.

We identified female pregnancy by visual swelling of the abdomen and confirmed this by palpation and ultrasound scans during trapping [42]. Births occur overnight in an underground den, and were identified by the absence of pregnant females the following morning and a subsequent change in their body shape and mass loss [29,43]. Pups were first captured at emergence from the den, at around three to four weeks of age, weighed and sexed, and given a unique ID; see [44] for further details of the trapping procedure. When individuals were first trapped, a 2 mm^2^ skin sample was taken for extraction of DNA, which was used to construct a pedigree for assigning parentage. Parentage was assigned using MasterBayes 2.51 [45] and colony 2.0.5.7 [46] as described in [47], for a dataset of 2310 individuals born in the study area between the years 2000 and 2016. Lifetime reproductive success was determined as the total number of pups assigned to each individual. For full details of DNA extraction, genotyping, parentage assignment and pedigree construction, see [47,48].

### (c) Measuring early-life care

Shortly after emergence from the den, pups form one-to-one caring relationships with particular adults known as ‘escorts’, which feed, carry, groom and protect the pup from predators [36]. The majority of pups have an exclusive relationship with a single escort; where pups have multiple escorts, they spend the great majority of their time with a single ‘primary’ escort [28]. Escorting starts at around four weeks of age and continues until pups reach nutritional independence, when they are around 90 days old (hereafter defined as the ‘escorting period’). While pup–escort dyads are forming, pups aggressively defend access to their escort [49], but thereafter both parties (escort and pup) actively seek each other out to maintain the association [50]. Experiments demonstrate that escorts and pups can recognize each other's calls, and that escorts are particularly reactive to the distress calls of the specific pup in its care [50,51].

We observed escorting behaviour in 120 communal litters in 12 social groups that inhabited the study area between 2000 and 2016. Groups were visited an average of 12 times during the escorting period, for a minimum of 20 min (the duration of one pup focal observation session). Only those litters for which we had five or more observation sessions (on different days) were included in the analyses. Pup focals were conducted so that each pup was followed for 20 min, and at each minute interval, individuals within 30 cm of the focal individual were noted (focals were paused if the focal pup went out of sight, and resumed once sighted again). If the pup spent more than half of the 20 min focal within 30 cm of the same individual, that adult was marked as the escort for that focal session [27]. The proportion of the pup focals a pup was seen being escorted was taken as a measure of care it received, termed its ‘escorting index’. Consequently, the escorting index varies from 0 (never observed being escorted) to 1 (always observed being escorted).

### (d) Body mass and ecological data

The emergence body mass of pups was recorded when the pups were first trapped at three to four weeks of age; see above. Adult body mass measurements were collected as part of the group visits. Most individuals are trained to step onto portable weighing scales in return for a small milk reward and were weighed weekly in the morning before foraging started.

Climate data were collected by Mweya meteorological station, and after 2014 by the Banded Mongoose Research Project. Cumulative rainfall during the month before the litter was born was used as a proxy of resource availability, as previous studies indicate that rainfall in the previous 30 days is positively correlated with adult daily body mass gain and pregnancy rate [52,53]).

### (e) Statistical analyses and model selection

#### (i) Immediate survival and post-escorting survival to 1 year

We used generalized linear mixed models (GLMMs) with a binomial error structure and logit link function, to analyse predictors of survival to nutritional independence at three months, and survival to maturity at 1 year. Predictor variables were escorting index, emergence weight of the pup, cumulative rainfall in the month before birth, and sex of the pup. An interaction between sex and escorting index was included to test for differential effects of escorting between the sexes. Social group ID and communal litter ID into which the pup was born were included as random factors in the analyses. This allows the intercept of the model to vary by litter ID and group, to control for group-level and litter-specific factors.

#### (ii) Body mass

We used a linear mixed model (LMM) to look at predictors of body mass at 1 year. The model included predictor and random factors as above.

#### (iii) Age at maturity

We used LMMs to investigate the age at which first signs of reproductive activity were observed in females (first oestrus), and males (the first mate guarding or ‘pestering’ behaviour during group oestrus [33]). As the definition for the start of reproduction is different, the sexes were analysed separately, but otherwise both models included predictor and random factors as above (escorting index, emergence weight of the pup, rainfall during month before birth as predictors, and social group and litter as random factors).

#### (iv) Adult lifespan

Rainfall, weight at emergence, escorting index, sex of the individual and the interaction between sex and escorting index were included as predictors in an LMM of total lifespan, and litter and pack included as random factors.

#### (v) Lifetime reproductive success

In analyses of lifetime reproductive success, the total number of offspring was first fitted as the response variable in a GLMM with a Poisson error structure and a log link function. The sexes were analysed separately to improve model convergence. Emergence weight, escorting index, rainfall and weight at maturity were included as predictors, and litter and group as random factors. We then fitted the same models again, but using the log (total lifespan of the individual) as an offset in the model, to analyse whether the included variables predicted the *rate* at which individuals produced offspring by accounting for differences in lifespan.

In all analyses, weights and rainfall were standardized by subtracting the mean and dividing by standard deviation, to improve model convergence. The correlation of predictor variables in each analysis was checked to confirm that it was not high enough to cause model fitting issues [54]. Non-significant interactions were dropped to allow significance testing of main terms [55], but models were not simplified further [56]. In the analyses that involved fitting models with a normal error structure (body mass, age at maturity and adult lifespan), we visually checked the residuals to ensure they met the model assumptions of normally distributed residuals with homogeneous variance. Where necessary, we log-transformed the response variable (adult lifespan) to meet these assumptions.

Statistical analyses were done in R version 3.3.1 [57] and GLMM models fitted using R package lme4 [58]. The significance of predictor variables was determined by performing likelihood ratio tests comparing the full model with a model without the predictor variable, removing non-significant interactions to allow the main effects of variables involved in these interactions to be assessed [59]. We report the *χ*^2^ statistics and parameter estimates (*β* ± s.e.) for significant terms, and the full analysis results including non-significant parameter estimates are presented in the electronic supplementary material, tables S1–S3.

## 3. Results

### (a) Developmental impacts of early-life care

#### (i) Immediate survival

Pups that received more care were more likely to survive until nutritional independence, as were those that were heavier at emergence (binomial GLMM: emergence weight: *β* = 0.83 ± 0.15, $\chi_{1}^{2}=36.44,$ *p* < 0.00001; electronic supplementary material, table S1). Pup survival was higher in periods of higher rainfall (*β* = 0.51 ± 0.18, $\chi_{1}^{2}=8.66,$ *p* = 0.003), whereas the sex of the pup had no effect (*β* = −0.16 ± 0.22, $\chi_{1}^{2}=0.57,$ *p* = 0.45).

#### (ii) Post-escorting survival to 1 year

Beyond the escorting period, early-life care received did not predict survival to maturity at 1 year of age (binomial GLMM: escorting index: *β* = 0.11 ± 0.43, $\chi_{1}^{2}=0.06,$ *p* = 0.802; electronic supplementary material, table S1). Males were more likely to survive to maturity (*β* = 0.50 ± 0.24, $\chi_{1}^{2}=4.38,$ *p* = 0.036), whereas rainfall had no effect (*β* = 0.21 ± 0.14, $\chi_{1}^{2}=2.05,$ *p* = 0.152).

#### (iii) Body mass at maturity

**Figure 1.**
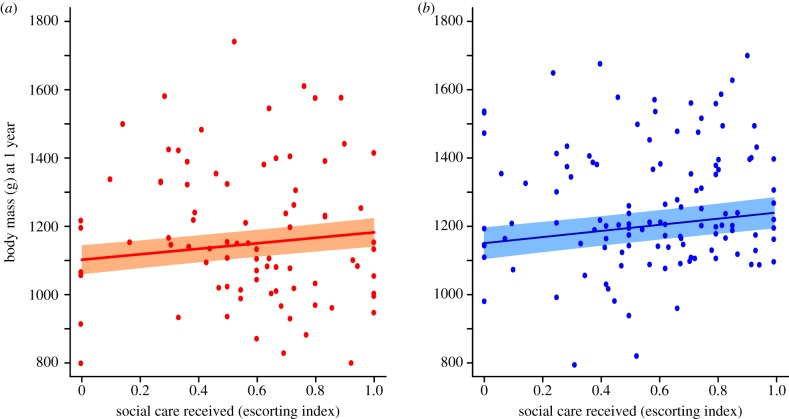
Escorting and body mass at sexual maturity (1 year). For both females and males, pups that were more closely escorted by adult helpers from days 30 to 90 of development were heavier at sexual maturity (1 year). Body mass at maturity is a positive predictor of lifetime reproductive success in both sexes. (*a*) Females; (*b*) males. Model prediction ± s.e. from a GLMM with litter and social group as random terms. Data from 203 individuals from 82 litters in 11 groups. (Online version in colour.)

Pups that received more care during the escorting period were heavier at 1 year of age, as were those that were heavier at emergence (figure 1; LMM, pups that survived to 12 months only: escorting index: *β* = 89.0 ± 33.5, $\chi_{1}^{2}=6.85,$ *p* = 0.009; emergence weight: *β* = 52.7 ± 11.8, $\chi_{1}^{2}=18.9,$ *p* < 0.001; for full model details, table 1).

**Table 1.** Predictors of body mass (in grams) at sexual maturity (1 year). Results from GLMMs with litter and social group as random factors. Raw data are grouped into mean for clarity. For categorical fixed factors, parameter estimates show the estimated difference between the level in [brackets] and the level represented by the intercept. Non-significant interactions were dropped to allow significance testing of main terms, but models were not simplified further. To improve model convergence, pup weight and rainfall were standardized (std) by subtracting the mean and dividing by the standard deviation.

body mass (g) at maturity, at 1 year of age			
fixed effects	*β* ± s.e.	$\chi_{1}^{2}$	*p*-value
(intercept)	1102.57 ± 47.50		
rainfall (std)	6.53 ± 12.04	0.304	0.581
body mass at emergence (std)	52.66 ± 11.75	18.90	1.378 × 10^−5^
escorting index	89.04 ± 33.53	6.846	0.0089
sex [male]	57.23 ± 18.58	9.439	0.0021
sex × escorting index	42.32 ± 64.97	0.434	0.510
number of observations	203 individuals, 82 litters, 11 packs		

#### (iv) Age at maturity

Female pups that received more care had their first oestrus earlier (LMM, pups that survived to 12 months only: escorting index: *β* = −0.34 ± 0.15, $\chi_{1}^{2}=5.16,$ *p* = 0.023). None of the tested variables predicted the timing of first observed mate guarding behaviour in males (all *p* > 0.2, see electronic supplementary material, table S2).

### (b) Lifetime impacts of early-life care

#### (i) Effects on adult lifespan

Adult lifespan was not longer for individuals that received more care as pups (escorting index: *β* = −0.03 ± 0.13, $\chi_{1}^{2}=0.06,$ *p* = 0.81; all other variables *p* > 0.098, see electronic supplementary material, table S3).

#### (ii) Effects on lifetime reproductive success

**Figure 2.**
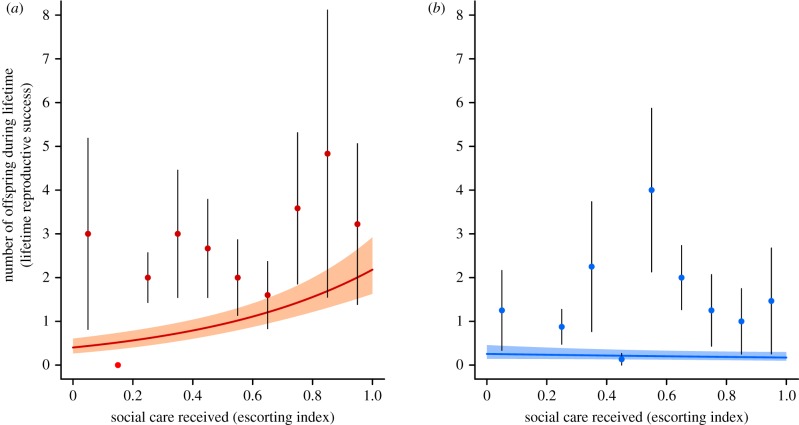
Escorting and lifetime reproductive success. (*a*) In females, increased escorting was associated with higher lifetime reproductive success as adults, independent of the positive effect of escorting on adult body mass. (*b*) In males, there was no additional effect of escorting *per se, c*ontrolling for effects on body mass. Model prediction ± s.e. from a GLMM with litter and social group as random terms. Data from 76 individuals from 55 communal litters in eight groups. Raw data grouped into mean ± s.e. for 10 equal bins for clarity. (Online version in colour.)

Females that received more care as pups had higher lifetime reproductive success (figure 2*a*; escorting index: *β* = 1.691 ± 0.506, $\chi_{1}^{2}=12.39,$ *p* = 0.0004), as did those that experienced heavier rainfall during the first month of life (*β* = 0.51 ± 0.24, $\chi_{1}^{2}=4.91,$ *p* = 0.027) and that were heavier at maturity (weight at 1 year: *β* = 0.48 ± 0.20, $\chi_{1}^{2}=4.3,$ *p* = 0.038). When using lifespan as an offset, the amount of care and weight at 1 year were the only significant predictors of a female's lifetime reproductive success (table 1). Thus, female pups that received more care in early life had greater lifetime reproductive success because they produced surviving offspring at a higher rate across their lifespan, not because they lived longer. Of the female pups that survived to adulthood, those that had been lighter at emergence had higher lifetime reproductive success (*β* = −0.46 ± 0.19, $\chi_{1}^{2}=5.69,$ *p* = 0.017; although not when using lifespan as an offset; see table 2 and electronic supplementary material, table S3). This unexpected finding may reflect selective disappearance during development (e.g. [60]): most lightweight pups die before reaching adulthood, so those lightweight pups for which we have a measure of lifetime reproductive success may represent a special subset of high-quality or high-survivorship individuals, compared with pups for which early-life mortality is less severe (electronic supplementary material, table S1).

**Table 2.** Predictors of lifetime reproductive success in individuals that reached maturity (lifespan > 365 days). Results from GLMMs with litter and social group as random factors. To improve model convergence, rainfall, mass at emergence and mass at maturity were standardized by subtracting the mean and dividing by the standard deviation.

	females			males		
fixed effects	*β* ± s.e.	$\chi_{1}^{2}$	*p*-value	*β* ± s.e.	$\chi_{1}^{2}$	*p*-value
(*a*) predictors of lifetime reproductive success: model without offset						
(intercept)	−0.695 ± 0.414			−1.276 ± 0.592		
rainfall (std)	0.506 ± 0.237	4.905	0.027	−0.349 ± 0.350	0.976	0.323
mass at emergence (std)	−0.461 ± 0.189	5.685	0.017	0.115 ± 0.235	0.238	0.626
escorting index	1.691 ± 0.506	12.388	0.0004	−0.392 ± 0.560	0.491	0.483
mass at 1 year (std)	0.476 ± 0.197	4.321	0.038	0.746 ± 0.233	11.027	0.0009
number of observations	76 individuals, 55 litters, 8 groups			109 individuals, 61 litters, 9 groups		
(*b*) predictors of lifetime reproductive success: model using lifespan as an offset						
(intercept)	−1.094 ± 0.346			−1.989 ± 0.481		
rainfall (std)	0.310 ± 0.185	2.972	0.085	−0.342 ± 0.269	1.588	0.208
mass at emergence (std)	−0.343 ± 0.160	2.596	0.107	−0.219 ± 0.218	1.016	0.313
escorting index	0.859 ± 0.436	4.122	0.042	−0.276 ± 0.552	0.251	0.617
mass at 1 year (std)	0.533 ± 0.152	4.934	0.026	0.508 ± 0.216	5.523	0.019
number of observations	76 individuals, 55 litters, 8 groups			109 individuals, 61 litters, 9 groups		

In males, there was no significant effect of early-life care on lifetime reproductive success (figure 2*b*; *β* ± s.e. =−0.39 ± 0.56, $\chi_{1}^{2}=0.49,$ *p* = 0.48). The only significant predictor of male lifetime reproductive success was body mass at 1 year, with males that were heaviest at maturity gaining highest lifetime reproductive success (*β* ± s.e. = 0.75 ± 0.23, $\chi_{1}^{2}=11.03,$ *p* = 0.0009; all other variables *p* > 0.3: table 2). Results were similar when using lifespan as an offset, and the only significant predictor of male lifetime reproductive success was mass at 1 year (table 2).

## 4. Discussion

Our results suggest that early-life care directed by escorts to specific offspring has both immediate survival benefits and durable fitness benefits that are manifested across the offspring's subsequent lifespan. The immediate survival benefits are expected because adult escorts and pups stay in close proximity throughout the day, and escorts are quick to alert, defend and carry their pup away from danger. The durable fitness benefits of being escorted are striking and manifested in two ways. First, for both male and female pups, escorting had a durable impact on body mass at maturity, which is positively associated with lifetime reproductive success in both sexes. In addition, independent of any effect on body mass, female pups that received higher levels of escorting were more efficient at producing surviving offspring and had higher lifetime reproductive success compared with females that received little escorting (table 2).

The presence of these durable fitness benefits to the recipients of early-life care is consistent with numerous findings from laboratory studies which suggest that the quality of parental care received in early life can have a profound impact on adult physiology, health and behaviour. In a classic laboratory study of Long–Evans hooded rats, offspring that received more licking and grooming from their mothers in the first 10 days of life showed reduced hypothalamic–pituitary–adrenal (HPA) endocrinological stress reactivity as adults [61]. Moreover, those (female) offspring were also more likely to express high levels of nurturing behaviour when they became mothers themselves, suggesting that early-life care can produce a chain of behavioural effects and potential benefits to recipients that last generations into the future. The transgenerational inheritance of grooming/licking behaviour in rats has a well-established epigenetic basis [62]. If such mechanisms operate in natural populations, cooperative care directed at offspring could have self-reinforcing or even runaway effects on levels of local helping (in the case where helped offspring are more likely to provide help themselves), or self-limiting effects (if helped offspring are less likely to provide help at a later date). The transgenerational impacts of cooperation are rendered plausible by the detailed mechanistic work on laboratory rodents, and are a fruitful area for both theoretical and empirical research. One of our future aims is to use the unusual escort system to investigate possible transgenerational influences on individual cooperative behaviour in this system.

In our study, only female offspring experienced an additional lifetime fitness benefit of being escorted *per se*, over and above any effect on body mass. This sex difference may reflect differences in the sensitivity of female and male reproductive systems to the conditions experienced in development, or sex differences in the key physical attributes (e.g. body size versus stress physiology) linked to reproductive success. It may also reflect a unisexual pattern of epigenetic inheritance of maternal-care-like behaviour. In the rat studies, both male and female offspring showed similar impacts of being licked/groomed on HPA reactivity and development, but only mothers provide care in this system, and hence only daughters inherited an elevated propensity to lick/groom their own offspring [63]. A third factor in banded mongooses is that there is a sex difference in the time delay to the realization of any durable benefit: males form a strict dominance hierarchy and must wait much longer to start reproducing compared with females (3+ years versus 1 year for females; [33]), so any durable benefits of being escorted as a pup may become diluted by other factors (environmental and/or social) that impinge on male lifetime reproductive success in the interim.

Theoretical analyses of durable impacts of help have focused on external benefits that arise through niche construction or the production of durable physical objects and structures [25]. Our findings suggest that durable benefits can also arise through development, for example, because recipients of help are protected from external insults or stressors during sensitive developmental windows, or are able to carry over extra resources to adulthood [26]. Lehmann's [25] model predicts that where the benefits of help are separated in time from the act of helping, selection for helping is strengthened (other things being equal). Selection for helping is particularly strong where benefits are realized after the actor has died or ceased reproduction, and is therefore unable to experience any negative effects of the increase in local competition resulting from the helping act. Thus, we can predict that where helping results in ‘internal’ durable benefits, selection for helping should increase with helper age, because older helpers are less likely to suffer direct competition from offspring produced as a result of their help. In humans, killer whales and elephants, grandmothers have demonstrable positive impacts on the reproductive success of their offspring [10,11,64–66]. However, these and other studies typically assume that any benefits associated with grandmother presence cease upon her death, whereas our study suggests that the impact of care may persist long after a helper has died. Durable benefits might go some way towards explaining why, in humans, many analyses have found that the (immediate) measurable fitness benefits of grandmothering are too small to favour the evolution of menopause ([67,68]; but see [12]).

Both our study and studies of grandmothering are examples where it is natural to assume that the recipients of help are members of a younger generation, such as young offspring or younger breeders. By contrast, most studies of cooperative breeding focus on the impact of help on the reproductive success of breeding adults, rather than their offspring [32]. In principle, Hamilton's rule could be used to determine the direction of selection on genes in parents or in their offspring—what matters in each case is correct consideration of genetic relatedness and recipient reproductive value (e.g. [69]). In banded mongooses, it is natural to view individual offspring as the recipients of help, not their parents, because each offspring is the sole beneficiary of the care provided by escorts, while the other offspring of the parent are cared for by other individuals. In other cooperative breeders, it is more practical to focus on parental fitness because help is provided to multiple offspring at a time, and it is difficult to track the impact of help on the reproductive success of all these younger recipients across their life course. However, our study suggests that an exclusive focus on parental reproductive success (measured as their number of surviving young) does not take account of any durable benefits of help and hence may systematically underestimate the strength of selection for altruism in natural systems.

In conclusion, our multigenerational study of a cooperative mammal living in the environment in which it evolved suggests that helping has lifelong fitness impacts on both male and female offspring. These durable fitness benefits may be challenging to detect and measure, particularly in long-lived species. Nevertheless, the extensive literature on early-life effects gives reason to believe that durable impacts may be widespread and can be expected to have major impacts on social evolution and life history. Further theoretical research is needed to investigate when durable benefits will result in positive or negative feedback between care received and helping effort in cooperative societies, and the consequences for social evolution. Further empirical research is needed to test for these effects in wild animal societies, and to investigate whether such early-life effects in natural systems are mediated by epigenetic and neuroendocrinological changes similar to those observed in laboratory mammals.

## Supplementary Material

Vitikainen et al ESM Supplementary Tables

## Data accessibility

The data supporting the analyses are available as part of the electronic supplementary material.
