# Supplementary material for: Live long and prosper: durable benefits of early-life care in banded mongooses
Source: Philos Trans R Soc Lond B Biol Sci. 2019 Feb 25;374(1770):20180114. doi: 10.1098/rstb.2018.0114 (PMC6460079; doi:10.1098/rstb.2018.0114)
Supplement: Vitikainen et al ESM Supplementary Tables [file rstb20180114supp1.pdf]

SUPPLEMENTARY ONLINE MATERIAL

Live long and prosper: durable benefits of early life care in wild banded mongooses

Emma I. K. Vitikainen, Faye J. Thompson, Harry H. Marshall & Michael A. Cant

SUPPLEMENTARY TABLES

Table S1.

Developmental effects of care received during the escorting period (< 90 days of age), results from binomial GLMMs with litter and social group as random factors. (A) Immediate survival: the probability that a pup survives until nutritional independence at 90 days (B) Post-escorting survival: the probability that after the escorting period, a pup survives the period to maturity at one year of age. Nonsignificant interactions were dropped to allow significance testing of main terms, but models were not simplified further. For categorical fixed factors, parameter estimates show the estimated difference between the level in [brackets] and the level represented by the intercept. To improve model convergence, pup weight and rainfall were standardised by subtracting the mean and dividing by standard deviation.

(A) Predictors of immediate survival

Fixed effects	$\beta \pm$ SE	χ^2_1	*P*
(intercept)	-0.887 ± 0.268		
Rainfall (std)	0.511 ± 0.178	8.650	0.003
Sex of pup [male]	-0.160 ± 0.214	0.565	0.452
Weight at emergence (std)	0.834 ± 0.146	36.44	1.578×10^{-9}
Escorting index	2.700 ± 0.384	58.89	1.671×10^{-14}
Sex × Escorting index§	-0.725 ± 0.679	1.140	0.286
Number of observations	676 individuals, 120 litters, 11 packs		

(B) Predictors of post-escorting survival to one year

Fixed effects	$\beta \pm$ SE	χ^2_1	*P*
(intercept)	0.029 ± 0.307		
Rainfall (std)	0.206 ± 0.142	2.049	0.152
Weight at emergence (std)	0.086 ± 0.141	0.376	0.540
Escorting index	0.107 ± 0.425	0.063	0.802
Sex of pup [male]	0.497 ± 0.238	4.383	0.036
Sex × Escorting index§	-0.430± 0.833	0.265	0.607
Number of observations	365 individuals, 100 litters, 11 packs		

§ not included in final model

Table S2.

Developmental effects of care received during the escorting period (< 90 days of age): predictors of (a) weight at maturity and (b) age at first observed oestrus behaviour, results from GLMMs with litter and social group as random factors. For categorical fixed factors, parameter estimates show the estimated difference between the level in [brackets] and the level represented by the intercept. Nonsignificant interactions were dropped to allow significance testing of main terms, but models were not simplified further. To improve model convergence, pup weight and rainfall were standardised by subtracting the mean and dividing by standard deviation.

(A) Weight (g) at maturity, at one years of age

Fixed effects	$\beta \pm$ SE	χ^2_1	*P*
(intercept)	1102.57 ± 47.50		
Rainfall (std)	6.53 ± 12.04	0.304	0.581
Weight at emergence (std)	52.66 ± 11.75	18.90	1.378×10^{-5}
Escorting index	89.04 ± 33.53	6.846	0.0089
Sex [male]	57.23 ± 18.58	9.439	0.0021
Sex × Escorting index§	42.32 ± 64.97	0.434	0.510
Number of observations	203 individuals, 82 litters, 11 packs		

§ not included in final model

(B) Age (months) at first oestrus (females) or mate guarding/pestering during group oestrus (males)

	Females			*Males*		
Fixed effects	$\beta \pm$ SE	χ^2_1	*P*	$\beta \pm$ SE	χ^2_1	*P*
(intercept)	6.187 ± 0.145			6.232 ± 0.116		
Rainfall (std)	-0.068 ± 0.068	1.053	0.305	-0.078 ± 0.063	1.592	0.207
Weight at emergence (std)	0.032 ± 0.054	0.308	0.579	-0.001 ± 0.057	0.001	0.999
Escort index	-0.342 ± 0.145	5.160	0.023	0.026 ± 0.159	0.028	0.867
Number of observations	71 individuals, 51 litters, 8 packs			86 individuals, 52 litters, 9 packs		

Table S3: Lifetime impacts of early-life care. Predictors of (A) total lifespan (days, ln-transformed) and lifetime reproductive success (B) and (C), in individuals that reached maturity (i.e. lifespan > 365 days). Results from GLMMs with litter and social group as random factors. For categorical fixed factors, parameter estimates show the estimated difference between the level in [brackets] and the level represented by the intercept. Nonsignificant interactions were dropped to allow significance testing of main terms, but models not simplified further. To improve model convergence, weights and rainfall were standardised by subtracting the mean and dividing by standard deviation.

(A) Predictors of lifespan

Fixed effects	β ± SE	χ^2_1	*P*
(intercept)	6.945 ± 0.111		
Rainfall	-0.014 ± 0.042	0.119	0.730
Weight at emergence	0.004 ± 0.045	0.013	0.910
Escorting index	-0.028 ± 0.132	0.057	0.812
Sex [male]	0.049 ± 0.075	0.393	0.531
Sex × Escorting index§	-0.426 ± 0.260	2.722	0.098
Number of observations	212 individuals, 85 litters, 11 packs		

§ not included in the final model

(B) Predictors of lifetime reproductive success: model without offset

	Females			*Males*		
Fixed effects	β ± SE	χ^2_1	*P*	β ± SE	χ^2_1	*P*
(intercept)	-0.695 ± 0.414			-1.276 ± 0.592		
Rainfall (std)	0.506 ± 0.237	4.905	0.027	-0.349 ± 0.350	0.976	0.323
Weight at emergence (std)	-0.461 ± 0.189	5.685	0.017	0.115 ± 0.235	0.238	0.626
Escorting index	1.691 ± 0.506	12.388	0.0004	-0.392 ± 0.560	0.491	0.483
Weight at one year (std)	0.476 ± 0.197	4.321	0.038	0.746 ± 0.233	11.027	0.0009
Number of observations	76 individuals, 55 litters, 8 packs			109 individuals, 61 litters, 9 packs		

(C) Predictors of lifetime reproductive success: model using lifespan as an offset

	Females			*Males*		
Fixed effects	β ± SE	χ^2_1	*P*	β ± SE	χ^2_1	*P*
(intercept)	-1.094 ± 0.346			-1.989 ± 0.481		
Rainfall (std)	0.310 ± 0.185	2.972	0.085	-0.342 ± 0.269	1.588	0.208
Weight at emergence (std)	-0.343 ± 0.160	2.596	0.107	-0.219 ± 0.218	1.016	0.313
Escorting index	0.859 ± 0.436	4.122	0.042	-0.276 ± 0.552	0.251	0.617
Weight at one year (std)	0.533 ± 0.152	4.934	0.026	0.508 ± 0.216	5.523	0.019
Number of observations	76 Individuals, 55 litters, 8 packs			109 Individuals, 61 litters, 9 packs		
